# Supplementary material for: CD36 inhibits β-catenin/c-myc-mediated glycolysis through ubiquitination of GPC4 to repress colorectal tumorigenesis
Source: Nat Commun. 2019 Sep 4;10:3981. doi: 10.1038/s41467-019-11662-3 (PMC6726635; doi:10.1038/s41467-019-11662-3)
Supplement: Supplementary file 1 — Supplementary Information [file 41467_2019_11662_MOESM1_ESM.pdf]

Supplementary Information

CD36 inhibits β-catenin/c-myc-mediated glycolysis through ubiquitination of GPC4 to repress colorectal tumorigenesis

Fang et al.

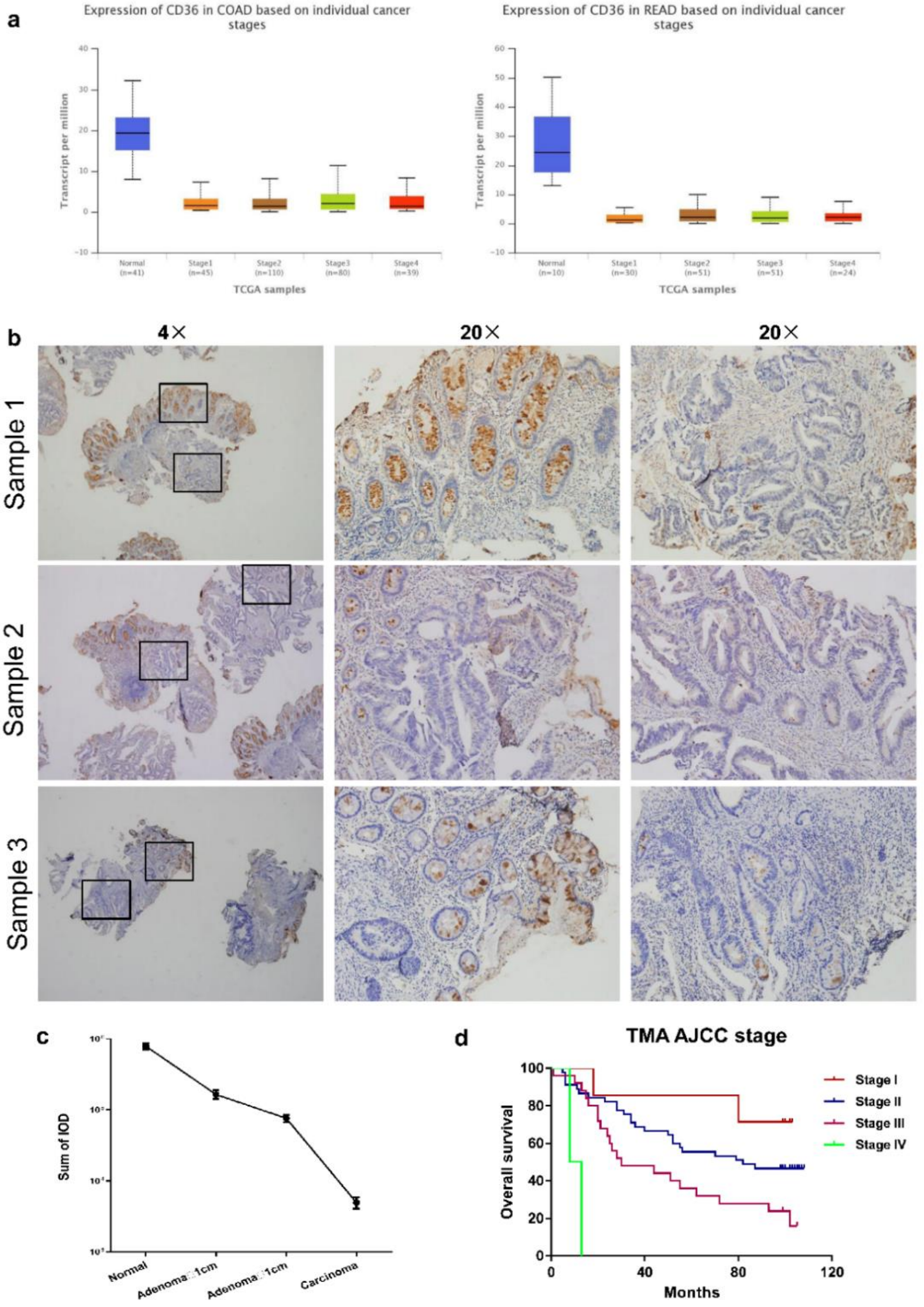

Supplementary Figure. 1 Loss of CD36 is associated with malignant transformation of human CRC development, related to Fig. 1 a CD36 mRNA level was down-regulated in all CRC stages as compared with normal tissues in the TCGA cohorts from UALCAN website. COAD, Colon Adenocarcinoma, READ, Rectal Adenocarcinoma. **b** Representative IHC staining images of adenomas with malignant transformation. **c** IHC staining of CD36 was progressively decreased from normal tissues, to adenomas and to carcinomas. **d** AJCC stage was a significant prognostic factor in the TMA. Source data are provided as a Source Data file.

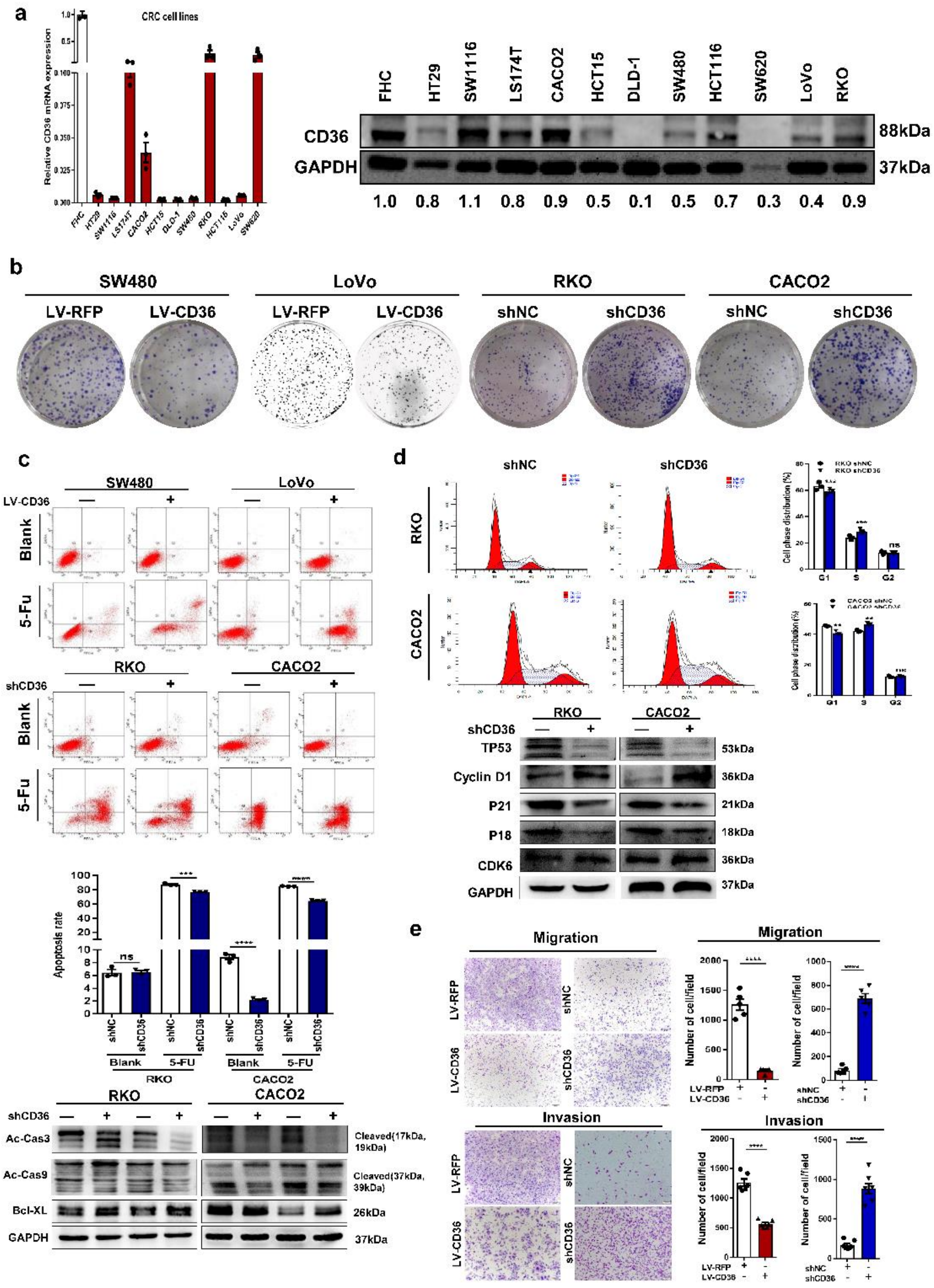

Supplementary Figure. 2 CD36 plays anti-carcinogenic roles in CRC cells, related to Fig. 2 a qRT-PCR and Western blots analysis of the mRNA and protein expression of CD36 in normal colonic mucosa cell line FHC and different CRC cell lines. **b** Images of colony formation in indicated cell lines, related to Fig. 2a. **c** Images of apoptosis in indicated cell lines under normal or 5-Fu treated conditions, related to Fig. 2b (up). Cell apoptosis was measured in RKO and CACO2 (shNC vs. shCD36) cell lines and Western blots of apoptosis markers (down). **d** Cell cycle analysis and Western blots of cell cycle markers. **e** Transwell migration and invasion assays in indicated cell lines. Each experiment was performed in at least triplicate and results are presented as mean ± SEM. Student t test or 1-way ANOVA was used to analyze the data (***P* < .01, ****P* < .001, *****P* < .0001). Source data are provided as a Source Data file.

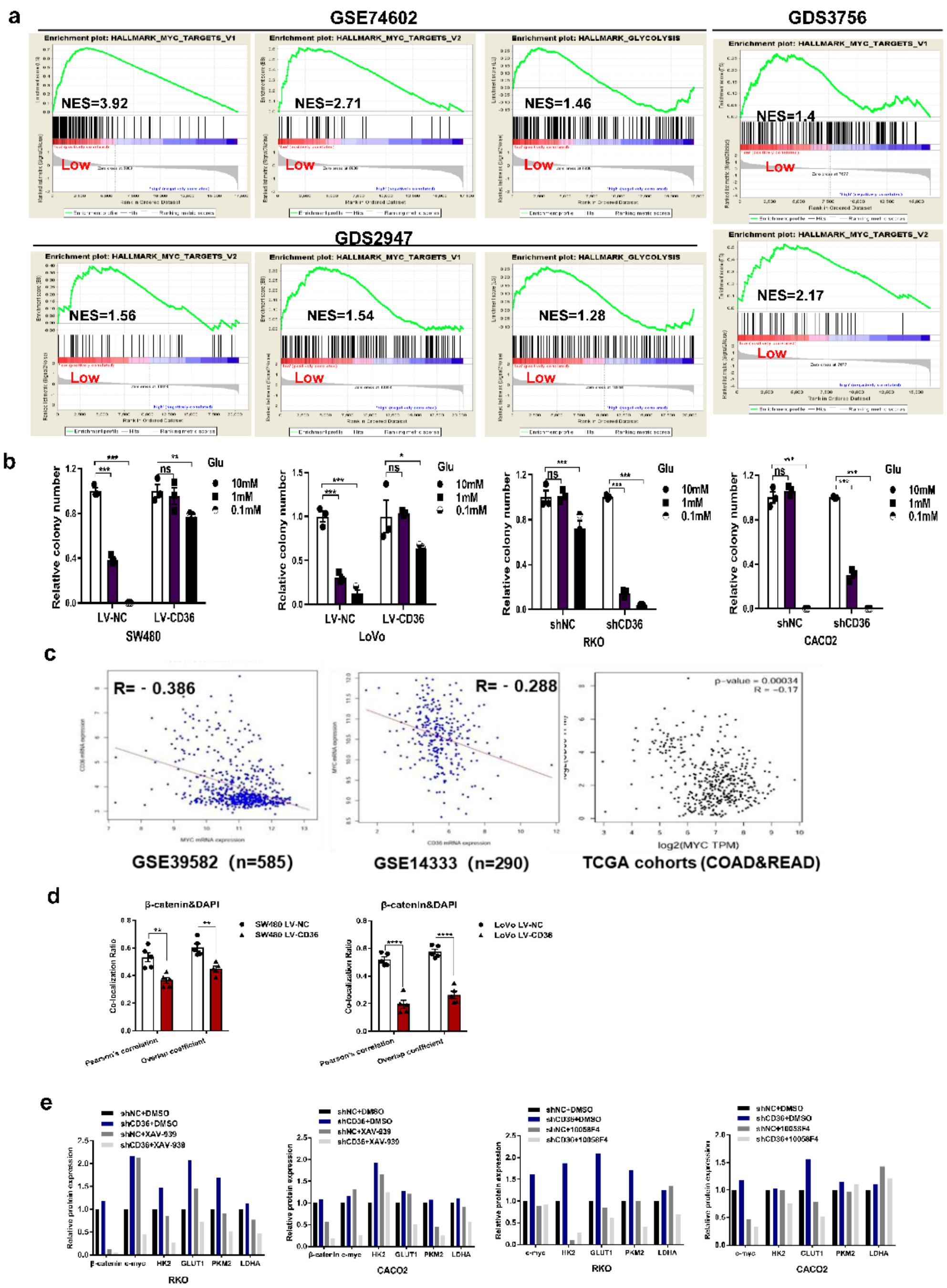

Supplementary Figure. 3 CD36 negatively correlates with c-myc signaling, related to Fig. 3 a GSEA hallmark analysis of GEO datasets (GSE74602, GDS2947, GDS3756), the mRNA data were arranged according to CD36 expression, and comparisons were between the top 25 percent and last 25 percent of CD36 expression. **b** Statistical results related to Fig. 3a, Results are shown as mean ± SEM (n=3), ***P* < .01, ****P* < .001, *****P* < .0001, based on 2-way ANOVA. **c** Correlation between CD36 and MYC mRNA levels in different GEO datasets (GSE39582, GSE14333, TCGA cohorts). **d** Statistical analysis of the co-localization of β-catenin and DAPI as shown in Fig. 3c. Results are shown as mean ± SEM (n=3), **P* < .05, ***P* < .01, ****P* < .001, *****P* < .0001, based on 2-way ANOVA. **e** Relative protein expression related to Fig. 3d.

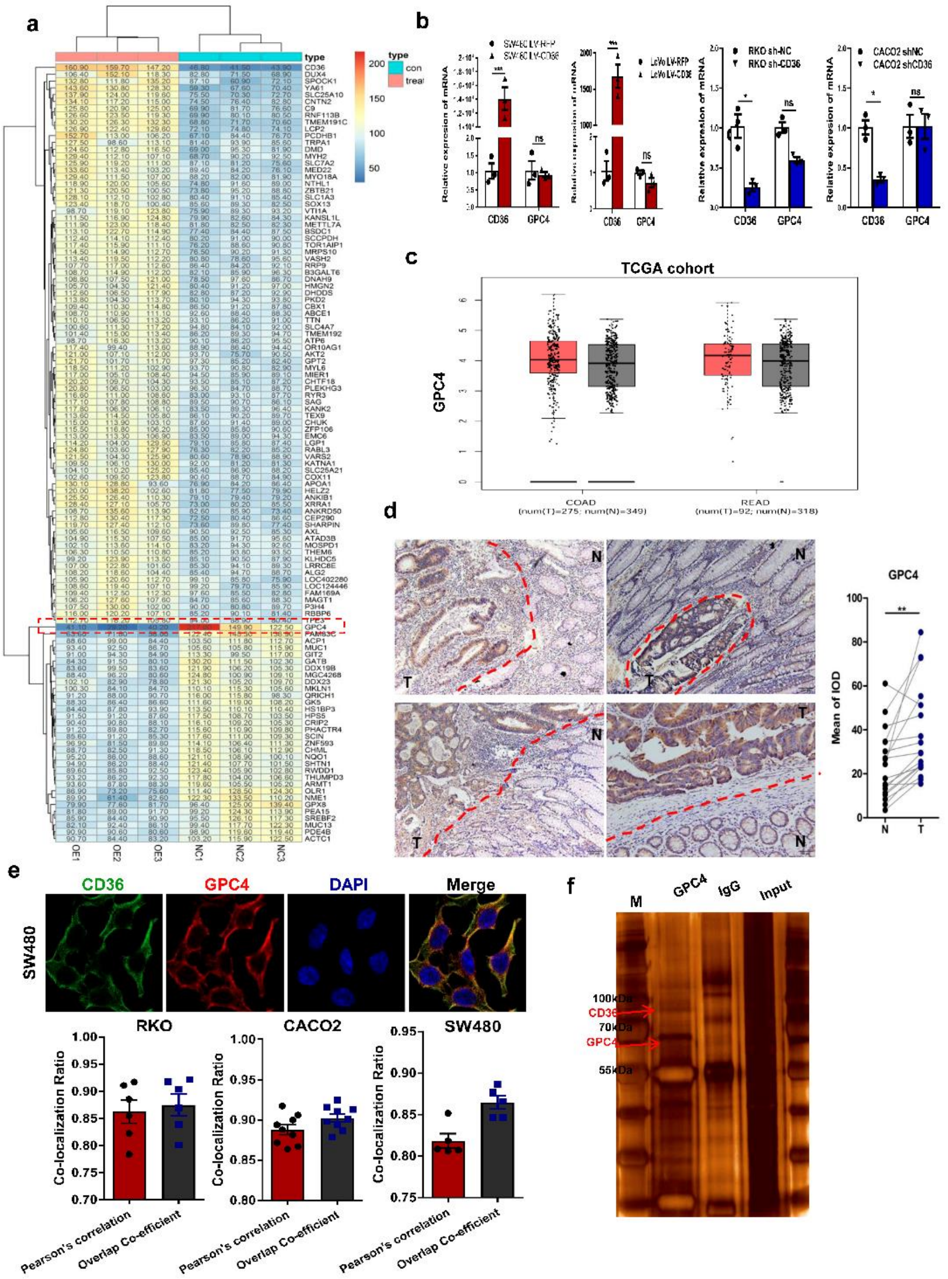

Supplementary Figure. 4 Protein expression of GPC4 in CRC and co-expression of GPC4 and CD36, related to Fig. 4. a Heat map of significantly changed proteins in comparison of SW480 LV-CD36 and LV-RFP cell lines. GPC4 was the most downregulated protein after CD36 overexpression. **b** mRNA levels of GPC4 in CRC cell lines with different CD36 expression. **c** GPC4 mRNA levels were compared in the TCGA cohorts. **d** IHC staining of GPC4 in transition zones of patients CRC tissues (left); Staining intensity of GPC4 was compared between normal and tumor locations in the transition zones. Results are shown as mean ± SEM, **P* < .05, ***P* < .01, ****P* < .001, *****P* < .0001, based on 2-way ANOVA or Student t test. **e** IF analysis of the co-localization of CD36 (green) and GPC4 (red) in SW480 cell lines (up) and the quantitative values of the co-localization of them related to Fig. 4b. **f** Anti-GPC4 and Anti-IgG co-immunoprecipitated proteins and the whole lysates were stained by silver staining. Source data are provided as a Source Data file.

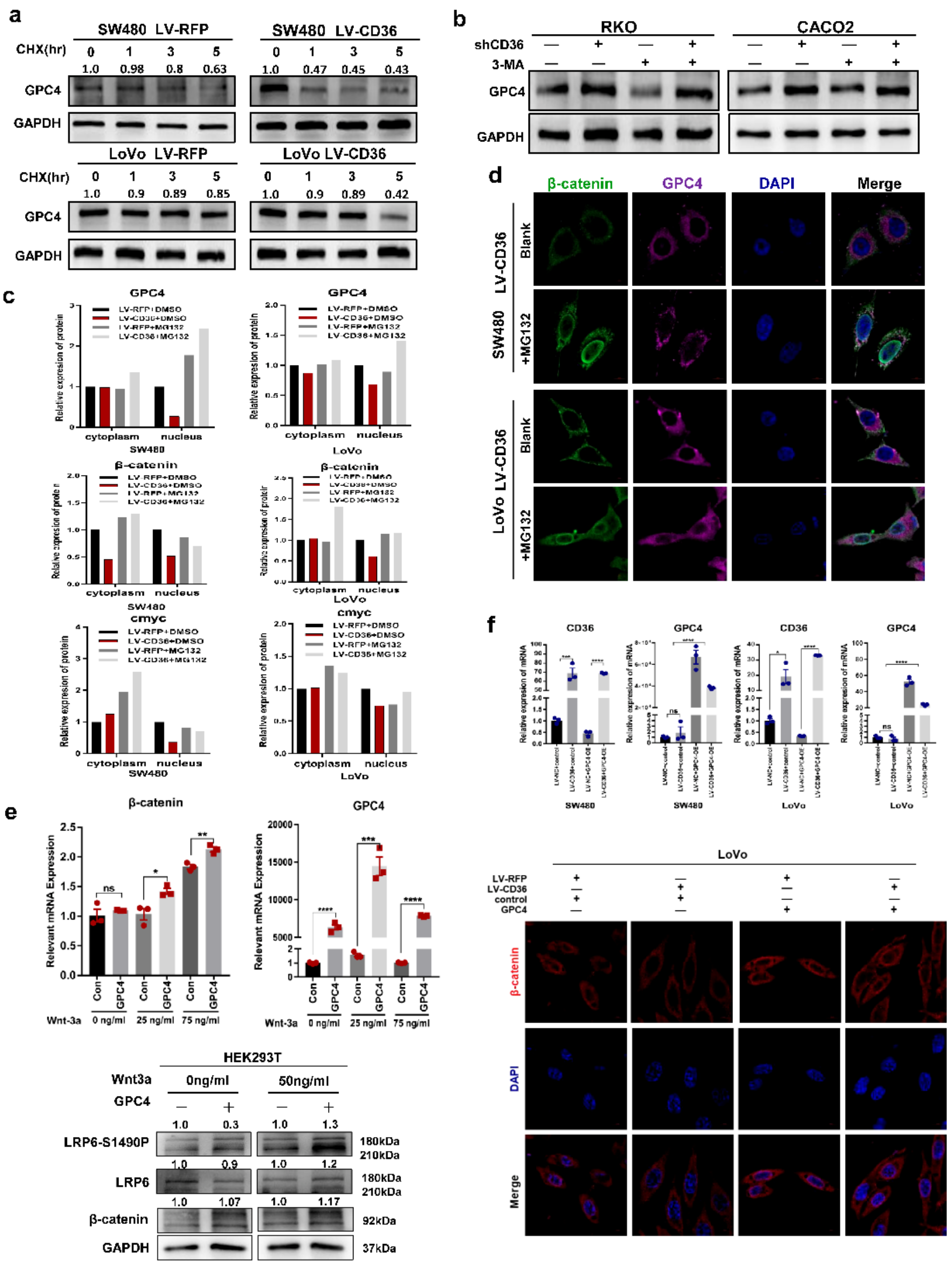

Supplementary Figure 5. GPC4 is an indispensable downstream of CD36, related to Fig. 4 and Fig. 5. a CHX (20 µg/ml) treatment to compare the stability of GPC4 in CD36-overexpressed or control cells. **b** 3-MA (a lysosome inhibitor, 20µM, 24h) treatment. **c** Relative cytoplasmic and nuclear protein expressions related to Fig. 5a. **d** IF analysis of the location of β-catenin with/without MG132 treatment. **e** Identification of the mRNA level of GPC4 and β-catenin after Wnt3a treatment in GPC4-overespressed and control 293T cells. Western blot analysis of indicated proteins in Wnt3a treated GPC4-overexpressed 293T cells. **f** Identification of the transfection efficiency of GPC4 by qRT-PCR, and IF analysis of β-catenin location after forced expression of GPC4 in LoVo cell lines. All statistics are shown as mean ± SEM (n=3), **P* < .05, ***P* < .01, ****P* < .001, *****P* < .0001, based on Student t test. Source data are provided as a Source Data file.

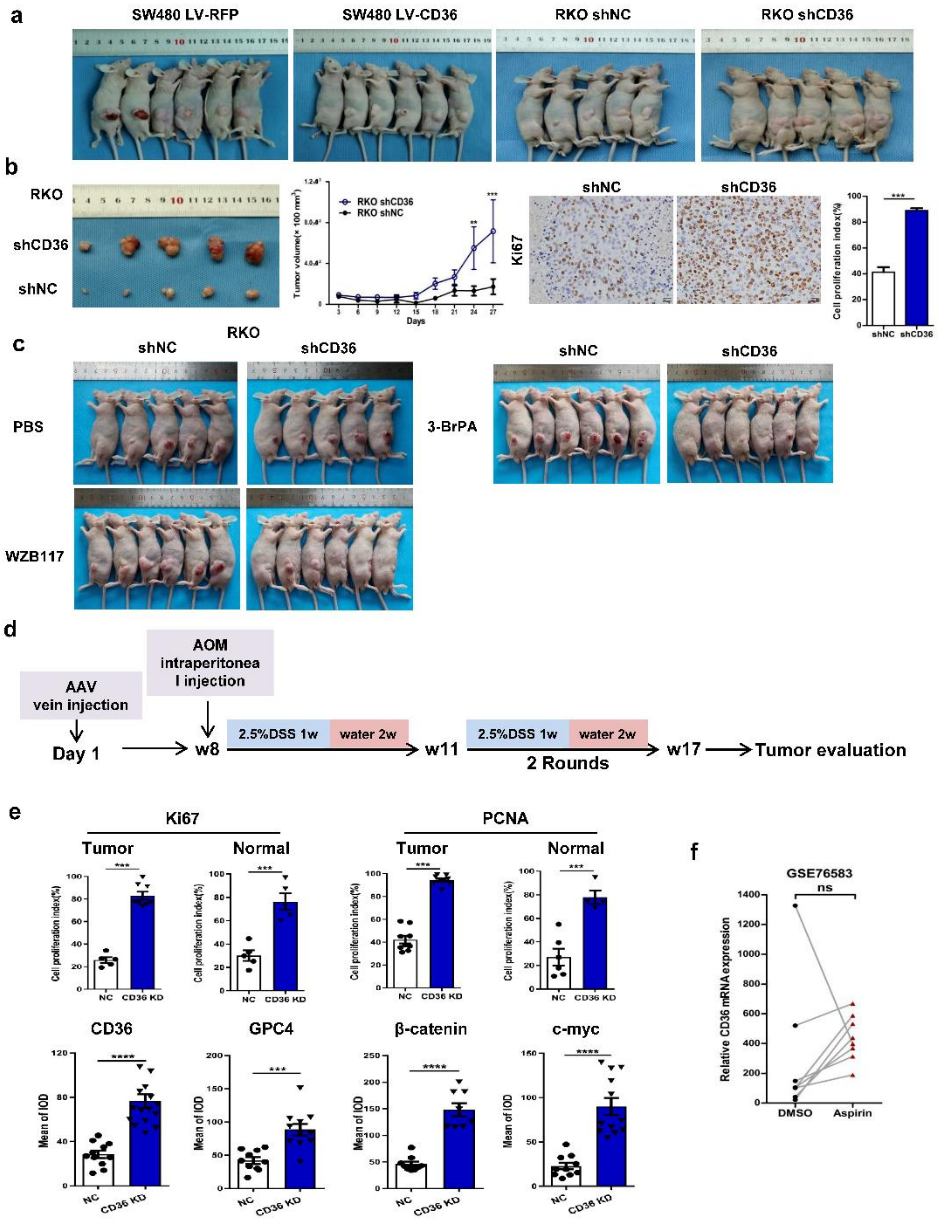

Supplementary Figure. 6 CD36 suppresses tumor growth in vivo, related to Fig. 6 and Fig. 7 a Macroscopic appearance of subcutaneous xenograft tumor growth in nude mice. **b** Tumor growth was measured and compared (left) in RKO (shNC vs. shCD36) cell lines. Cell proliferation was determined by Ki67 staining and cell proliferation index was quantified. **c** Macroscopic appearance related to Fig.6e. **d** Detailed induction procedures in AAV-transfected AOM/DSS-induced CRC models. **e** Statistics of staining intensity of Ki67, CD36, GPC4, β-catenin and c-myc related to Fig.7b. Results are shown as mean ± SEM, ****P* < .001, *****P* < .0001, based on Student t test or 2-way ANOVA. **f** CD36 mRNA levels was upregulated in 7 out of 8 CRC cells after treatment with aspirin in GSE76583, although the overall comparison was not significantly different. Source data are provided as a Source Data file.

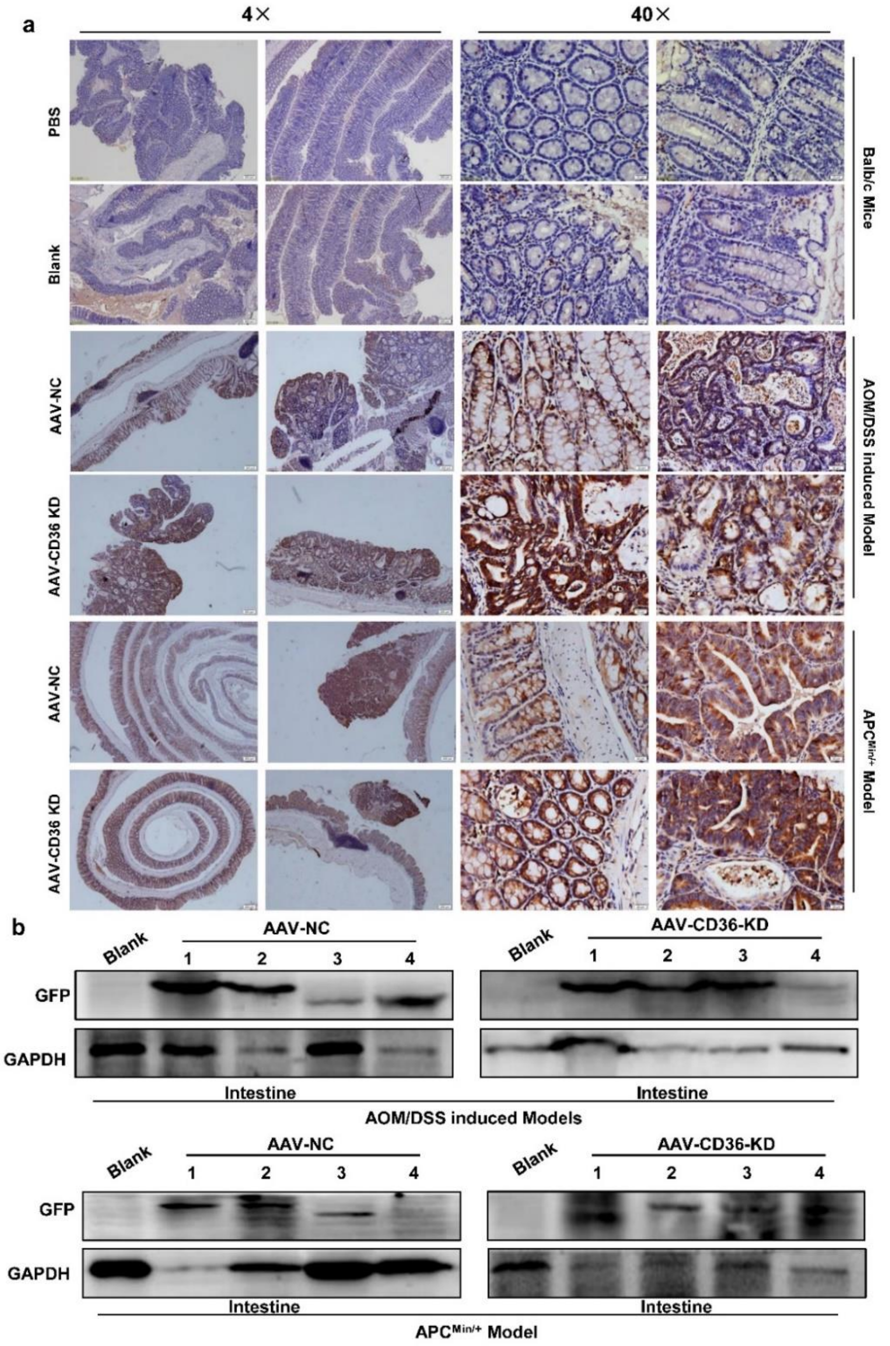

Supplementary Figure. 7 AAV transfection efficiency. a Immunohistochemistry (IHC) of GFP in the colon tissues of Balb/C mice without any treatment, AOM/DSS-induced mice models and *Apc*$^{Min/+}$ mice. **b** Western blots of GFP in intestine tissues of both AOM/DSS-induced mice and *Apc*$^{Min/+}$ mice. Source data are provided as a Source Data file.

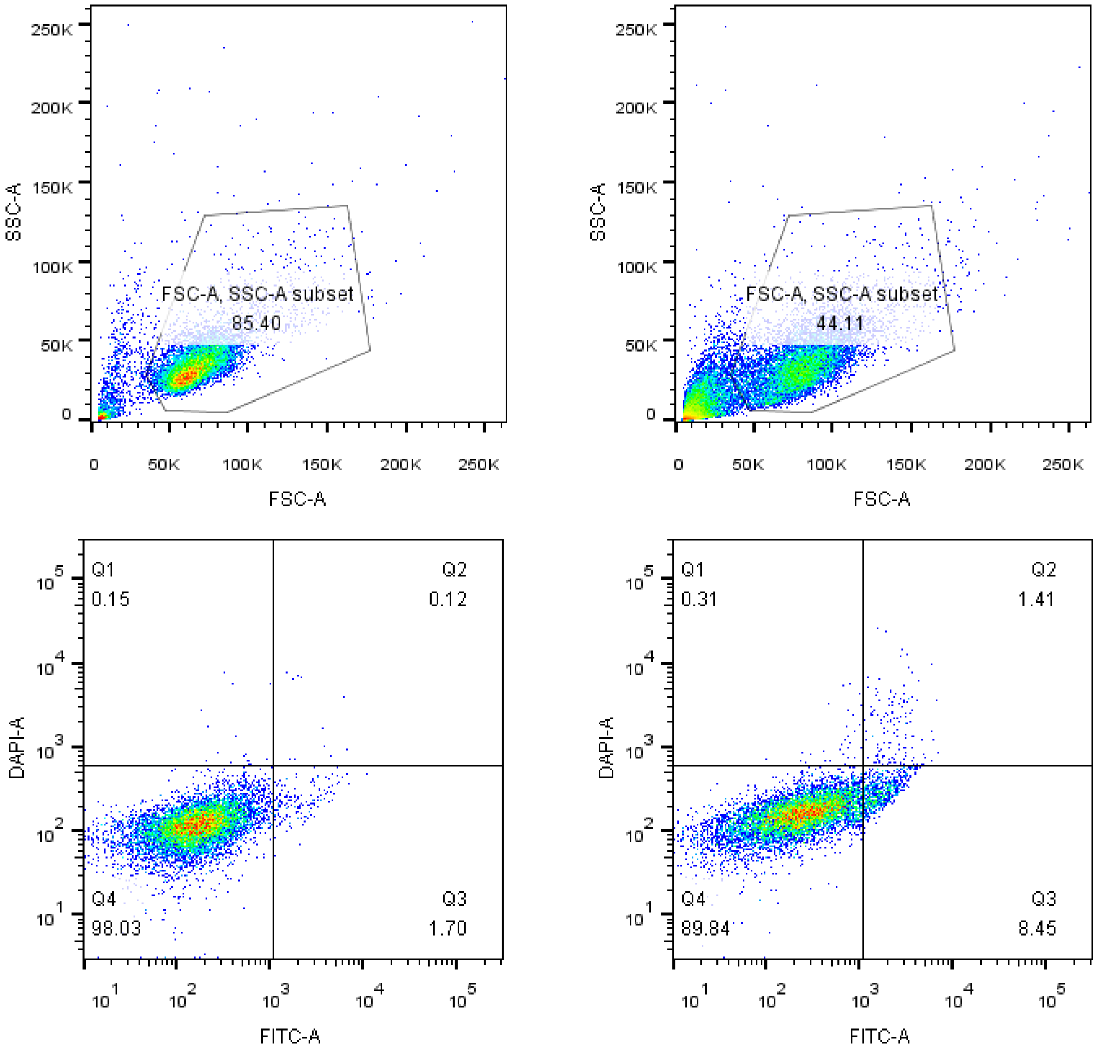

Supplementary Figure. 8 Flow cytometry gating strategy. Gating strategy in flow cytometry analysis to contain 1×10^4 non-debris starting events were represented. Small debrises were excluded on a Side-Scatter (Area) vs. Forward-Scatter (Area) dot plot. The data shown above were gated to contain non-debris singlets. The non-debris singlets populations comprised 85.40% of all events in control group and 44.11% in 5-Fu treatment group. The non-debris cells were analyzed on FITC (Area) vs. DAPI (Area) dot plot. And the boundaries between “positive” and “negative” staining cells were defined according to the control groups.

Supplementary Table 1. Correlation between CD36 expression and clinicopathologic characteristics of 81 patients with colon cancer, Source data are provided as a Source TMA file

Variables	All cases	CD36 expression		χ2	*P*-value
		Low (%)	High (%)		
**Gender**				1.766	0.184
**Male**	43	18(46.2)	15(61)		
**Female**	37	21(53.8)	16(39)		
**Age(years)**				1.162	0.281
**≤65**	28	11(30.6)	17(42.5)		
**> 65**	48	25(69.4)	23(57.5)		
**Tumor size(cm)[a]**				0.014	0.905
**≤5**	42	21(53.8)	21(52.5)		
**>5**	37	18(46.2)	19(47.5)		
**Differentiation**				0.11	0.917
**Well/moderate**	43	21(52.5)	22(53.7)		
**Poor**	38	19(47.5)	19(46.3)		
**Tumor invasion**				0.001	0.972
**Without breakthrough serosa**	63	29(85.3)	34(85.0)		
**Serosa or adjacent organs**	11	5(14.7)	6 (15.0)		
**Nodal metastasis**				1.580	0.209
**N0**	54	24(60.0)	30(73.2)		
**N1-2**	27	16(40.0)	11(26.8)		
**Distant metastasis**				2.102	0.147
**M0**	79	38(95.0)	41(100.0)		
**M1**	2	2(5.0)	0(0.0)		
**AJCC stage**				1.606	0.205
Ⅰ/ Ⅱ	52	23(59.0)	29(72.5)		
Ⅲ/ Ⅳ	27	16(41.0)	11(47.5)		

Supplementary Table 2. Univariate and multivariate Cox regression analyses of potential poor prognostic factors in colon cancer, Source data are provided as a Source TMA file

	Univariate		Multivariate	
**Variables**	**RR (95% CI)**	**p Value**	**RR (95% CI)**	**p Value**
**Gender**	1.037 (0.585 to 1.84)	0.901		
**Age**	2.21 (1.142 to 4.276)	**0.019**	1.904 (0.98 to 3.698)	0.057
**Differentiation**	1.38 (0.782 to 2.433)	0.266		
**Tumor size**	0.929 (0.522 to 1.652)	0.801		
**TNM stage**				
**T**	2.252 (1.243 to 5.128)	**0.02**		
**N**	2.482 (1.399 to 4.404)	**0.002**		
**M**	11.035 (2.359 to 51.615)	**0.002**		

**AJCC stage**	2.359 (1.33 to 4.183)	**0.003**	2.0 (1.111 to 3.602)	**0.021**
**CD36 group**	0.513 (0.288 to 0.913)	**0.023**	0.476 (0.262 to 0.864)	**0.015**

Supplementary Table 3: Antibodies used in this study

Antibody	Company	Catalog No.	Dilutions
**β-catenin**	GeneTex	GTX101435	CHIP 1:20; IHC 1:500
**β-catenin**	Proteintech	51067 2-AP	WB 1:1000
**β-catenin**	Santa Cruz	sc-59737	IF 1:100
**c-myc**	ABclonal	A0309	WB 1:500
**c-myc**	Genetex	GTX103436	IF 1:250; IHC 1:500
**LDHA**	GeneTex	GTX101416	IHC 1:500
**LDHA**	Abclonal	A1146	WB 1:1000
**PKM2**	Abclonal	A0268	WB 1:1000; IHC 1:500
**CD36**	Abcam	ab78054	WB 1:500; IHC 1:200
**CD36**	Santa Cruz	sc-7309	IF 1:100; IP 1:50
**HK2**	Abclonal	A0994	WB 1:1000
**SLC2A1**	Abclonal	A6982	WB 1:500
**GLUT1**	Abcam	ab115730	IHC 1:500
**GAPDH**	Proteintech	10494-1-AP	WB 1:2000
**FLAG tag**	Proteintech	66008-2-Ig	WB 1:1000; IP 1:100
**KI67 (Human)**	CST	9449	IHC 1:800
**KI67 (Mouse)**	CST	12202	IHC 1:800
**PCNA**	Abclonal	A0264	IHC 1:800
**Lamin B**	Abclonal	A2452	WB 1:500
**Glypcian 4**	Abcam	ab168364	WB 1:500; IP 1:50
**Glypcian 4**	Proteintech	13048-1-AP	IF 1:100
**GFP tag**	Proteintech	50430-2-AP	IHC WB 1:500
**Cell Cycle Kit**	CST	9932	WB 1:1000
**cleaved caspase 3**	CST	9664	WB 1:1000
**cleaved caspase 9**	CST	20750	WB 1:1000
**Bcl-XL**	Abcam	ab32370	WB 1:1000
**FK2**	EMD Millipore	ST1200	WB 1:1000; IP 1:100
**Ubiquitin (linkage-specific K48)**	Abcam	ab140601	WB 1:1000
**LRP6**	CST	2560	WB 1:1000
**P-LRP6 (Ser1490)**	CST	2568	WB 1:1000
**IgG (Mouse)**	CST	5873	IP 1:1000
**IgG (Rabbit)**	Sigma-Aldrich	R3155	IP 1:000

Supplementary Table 4: Primers used in this study

Gene	Forward	Reverse
**List of primers used in qRT-PCR.**		
CD36	GGGAAAGTCACTGCGACATG	TGCAATACCTGGCTTTTCTCA
GLUT1	CGGGCCAAGAGTGTGTGCTAAA	TGACGATACCGGAGCCAATG
MYC	CACCACCAGCAGCGACTCT	GGCACCTCTTGAGGACCAGT
LDHA	GGCCTGTGCCATCAGTATCT	GGAGATCCATCATCTCTCCC
GPC4	CAACCACAGCAGCTGGCACTA	TTGCAAACGTTGCTCGGAAG
GFP	TGGAACTGGATGGCGATGTG	TGCTGCTTCATATGGTCTGG
HK2	CCAGTTCATTCACATCATCAG	CTTACACGAGGTCACATAGC
PKM2	GACTGCCTTCATTCAGACCCA	GGGTGGTGAATCAATGTCCAG
GAPDH	ACCACAGTCCATGCCATCAC	TCCACCACCCTGTTGCTGTA
β-catenin	AAAGCGGCTGTTAGTCACTGG	CGAGTCATTGCATACTGTCCAT
β-actin	CTGGCTCCTAGCACCATGAAGAT	GGTGGACAGTGAGGCCAGGAT
**$Apc^{Min}/^{+}$ mice identification (PCR)**		
MR0033	GCCATCCCTTCACGTTAG	
MR0034	TTCCACTTTGGCATAAGGC	
MR0758	TTCTGAGAAAGACAGAAGTTA	
**ChIP assay**		
MYC	GCTCTCCACTTGCCCCTTTTA	GTTCCCAATTTCTCAGCC
Cyclin D1	GACTACAGGGGAGTTTTGTTG	TCGGCTCTCGCTTCTGCTG
**The primers sequences for CD36 gene CDS amplification.**		
Primer-F	AGGTCGACTCTAGAGGATCCCGCCACCATGGGCTGTGACCGGAACTGTGG	
Primer-R	TCCTTGTAGTCCATACCGGTTTTTATTGTTTTCGATCTGCATGC	
**List of CD36 shRNAs coding sequences**		
	Target sequence	
shCD36-1	GACGTTAATCTGAAAGGAA	
shCD36-2	ATTGGTGATGAGAAGGCAA	
**The primers sequences for GPC4 gene CDS amplification.**		
Primer-F	ACGGGCCCTCTAGACTCGAGCGCCACCATGGCACGGTTCGGCTTGCCCGCGC	
Primer-R	TTAAACTTAAGCTTGGTACCTTAATGATGATGATGATGATGTCTCCACTCTCTCTGCATAACC	
