# Supplementary material for: CD36 inhibits β-catenin/c-myc-mediated glycolysis through ubiquitination of GPC4 to repress colorectal tumorigenesis
Source: Nat Commun. 2019 Sep 4;10:3981. doi: 10.1038/s41467-019-11662-3 (PMC6726635; doi:10.1038/s41467-019-11662-3)
Supplement: Supplementary file 2 — Reporting Summary [file 41467_2019_11662_MOESM2_ESM.pdf]

nature research

Corresponding author(s): DBPR

Last updated by author(s): Jul 5, 2019

# Reporting Summary

Nature Research wishes to improve the reproducibility of the work that we publish. This form provides structure for consistency and transparency in reporting. For further information on Nature Research policies, see Authors & Referees and the Editorial Policy Checklist.

## Statistics

For all statistical analyses, confirm that the following items are present in the figure legend, table legend, main text, or Methods section.

| n/a | Confirmed | |
|---|---|---|
| ☐ | ☒ | The exact sample size (*n*) for each experimental group/condition, given as a discrete number and unit of measurement |
| ☐ | ☒ | A statement on whether measurements were taken from distinct samples or whether the same sample was measured repeatedly |
| ☐ | ☒ | The statistical test(s) used AND whether they are one- or two-sided<br>*Only common tests should be described solely by name; describe more complex techniques in the Methods section.* |
| ☒ | ☐ | A description of all covariates tested |
| ☒ | ☐ | A description of any assumptions or corrections, such as tests of normality and adjustment for multiple comparisons |
| ☐ | ☒ | A full description of the statistical parameters including central tendency (e.g. means) or other basic estimates (e.g. regression coefficient) AND variation (e.g. standard deviation) or associated estimates of uncertainty (e.g. confidence intervals) |
| ☒ | ☐ | For null hypothesis testing, the test statistic (e.g. *F*, *t*, *r*) with confidence intervals, effect sizes, degrees of freedom and *P* value noted<br>*Give P values as exact values whenever suitable.* |
| ☒ | ☐ | For Bayesian analysis, information on the choice of priors and Markov chain Monte Carlo settings |
| ☒ | ☐ | For hierarchical and complex designs, identification of the appropriate level for tests and full reporting of outcomes |
| ☐ | ☒ | Estimates of effect sizes (e.g. Cohen's *d*, Pearson's *r*), indicating how they were calculated |

*Our web collection on statistics for biologists contains articles on many of the points above.*

## Software and code

Policy information about availability of computer code

| | |
|---|---|
| Data collection | The Gene Expression Omnibus (GEO) data and TCGA data referenced during the study are available in a public repository from the GEO website (https://www.ncbi.nlm.nih.gov/geo/), GEPIA website (http://gepia.cancer-pku.cn) and UALCAN website (http://ualcan.path.uab.edu/cgi-bin/ualcan-res.pl). |
| Data analysis | Statistical analyses were performed using GraphPad Prism 7 or ImageJ software or Image Pro Plus software. |

For manuscripts utilizing custom algorithms or software that are central to the research but not yet described in published literature, software must be made available to editors/reviewers. We strongly encourage code deposition in a community repository (e.g. GitHub). See the Nature Research guidelines for submitting code & software for further information.

## Data

Policy information about availability of data

All manuscripts must include a data availability statement. This statement should provide the following information, where applicable:

- Accession codes, unique identifiers, or web links for publicly available datasets
- A list of figures that have associated raw data
- A description of any restrictions on data availability

The Gene Expression Omnibus (GEO) data and TCGA data referenced during the study are available in a public repository from the GEO website (https://www.ncbi.nlm.nih.gov/geo/), GEPIA website (http://gepia.cancer-pku.cn) and UALCAN website (http://ualcan.path.uab.edu/cgi-bin/ualcan-res.pl). The authors declare that all the other data supporting the findings of this study are available within the article and its Supplementary Information files and from the corresponding author on reasonable request. The source data underlying all figures are provided as Source Data files.

# Field-specific reporting

Please select the one below that is the best fit for your research. If you are not sure, read the appropriate sections before making your selection.

☒ Life sciences ☐ Behavioural & social sciences ☐ Ecological, evolutionary & environmental sciences

For a reference copy of the document with all sections, see nature.com/documents/nr-reporting-summary-flat.pdf

# Life sciences study design

All studies must disclose on these points even when the disclosure is negative.

| | |
|---|---|
| Sample size | no statistical method was used to predetermine sample size |
| Data exclusions | no data was excluded from the analyses |
| Replication | All attempts at replication generated reliable and similar results. The reproducibility of all the experiments is described in the figure legends. In all cases results are expressed as mean ± standard error of mean(SEM). Significance was analyzed using Student's t test, 1-Way ANOVA or 2-Way ANOVA where a p value of less than 0.05 was considered statistically significant. All statistical calculations were performed by GraphPad Prism 7. |
| Randomization | The animals were grouped by the same age and gender, and were randomly allocated to experimental groups . |
| Blinding | The investigator was blinded to the treatment of mice and human participant characteristics during experiments. |

# Reporting for specific materials, systems and methods

We require information from authors about some types of materials, experimental systems and methods used in many studies. Here, indicate whether each material, system or method listed is relevant to your study. If you are not sure if a list item applies to your research, read the appropriate section before selecting a response.

## Materials & experimental systems

| n/a | Involved in the study | |
|---|---|---|
| ☐ | ☒ | Antibodies |
| ☐ | ☒ | Eukaryotic cell lines |
| ☒ | ☐ | Palaeontology |
| ☐ | ☒ | Animals and other organisms |
| ☐ | ☒ | Human research participants |
| ☒ | ☐ | Clinical data |

## Methods

| n/a | Involved in the study | |
|---|---|---|
| ☒ | ☐ | ChIP-seq |
| ☐ | ☒ | Flow cytometry |
| ☒ | ☐ | MRI-based neuroimaging |

## Antibodies

| | |
|---|---|
| Antibodies used | All antibodies are commercial. The reference and dilution is described in Supplementary Table 3. |
| Validation | All the antibodies were validated with preliminary experiments. We examined primary antibodies according to manuals, and got similar results with validation results on manufacturer's website or relevant citations. |

## Eukaryotic cell lines

Policy information about cell lines

| | |
|---|---|
| Cell line source(s) | human embryonic kidney 293Tcell line (HEK293T), Normal colon epithelial cell line (FHC) and CRC cell lines (HT29, SW1116, LS174T, CACO2, HCT15, DLD-1, SW480, RKO, HCT116, LoVo and SW620) were purchased from the American Type Culture Collection (ATCC). |
| Authentication | ATCC Cell Certification has been uploaded |
| Mycoplasma contamination | All cell lines tested negative for mycoplasma contamination. |
| Commonly misidentified lines (See ICLAC register) | No commonly misidentified cell lines were used. |

# Animals and other organisms

Policy information about studies involving animals; ARRIVE guidelines recommended for reporting animal research

| | |
|---|---|
| Laboratory animals | Male athymic nude mice (BALB/c-nu/nu, 4 weeks old) and male BALB/c mice (4 weeks old) were purchased from the animal center of Guangdong Province. The ApcMin/+ mice were purchased from the GENECHEM Biotech at Shanghai, all mouse care and experiments were approved by the Institutional Animal Care and Use Committee (IACUC) of Nanfang Hospital. |
| Wild animals | the study did not involve wild animals |
| Field-collected samples | the study did not involve samples collected from the field |
| Ethics oversight | All mouse care and experiments were approved by the Institutional Animal Care and Use Committee (IACUC) of Nanfang Hospital. |

Note that full information on the approval of the study protocol must also be provided in the manuscript.

# Human research participants

Policy information about studies involving human research participants

| | |
|---|---|
| Population characteristics | Fresh and formalin-fixed tissue samples from patients with colorectal neoplasia were collected randomly from the Department of Gastroenterology or the Department of General Surgery, Nanfang Hospital, affiliated to Southern Medical University, and detailed population characteristics were shown in Source data file. A tissue microarrays (TMA) derived from 90 patients' resections of CRC and distal normal mucosa were purchased from the National Engineering Center for Biochip at Shanghai. |
| Recruitment | A total of 129 specimens involved 75 patients (11 colonic lesions taken from the Department of Gastroenterology, 54 pairs CRC samples and matched normal mucosa and 10 transition zone of CRC samples collected from the Department of General Surgery) were collected from January 1, 2015 to January 1, 2018. |
| Ethics oversight | The Institute Research Medical Ethics Committee of Nanfang Hospital granted approval for this study. |

Note that full information on the approval of the study protocol must also be provided in the manuscript.

# Flow Cytometry

## Plots

Confirm that:

☒ The axis labels state the marker and fluorochrome used (e.g. CD4-FITC).

☒ The axis scales are clearly visible. Include numbers along axes only for bottom left plot of group (a 'group' is an analysis of identical markers).

☒ All plots are contour plots with outliers or pseudocolor plots.

☒ A numerical value for number of cells or percentage (with statistics) is provided.

## Methodology

| | |
|---|---|
| Sample preparation | Sample preparation is described in the Methods. |
| Instrument | BD LSRFortessa X-20 |
| Software | FlowJo |
| Cell population abundance | 5×103-1×104 cells were measured for analysis every time |
| Gating strategy | Representative gating strategy for excluding debris in flow cytometry analysis of 1×104 events. Small debris are excluded on a Side-Scatter (Area) vs. Forward-Scatter (Area) dot plot (a). Then the cell population was analysed by 2 fluorescence channels include FITC and DAPI (for apoptosis analysis) or 1 fluorescence channel (FITC for 2NBDG uptake or DAPI for cell cycle analysis). The boundaries between "positive" and "negative" staining cells were gated according to the control groups. Gating strategy is presented in Supplementary Figure 8. |

☒ Tick this box to confirm that a figure exemplifying the gating strategy is provided in the Supplementary Information.
